# Comparative Evaluation of Pellet Cushioning Agents by Various Imaging Techniques and Dissolution Studies

**DOI:** 10.1208/s12249-020-01902-x

**Published:** 2020-12-29

**Authors:** Konrád Sántha, Nikolett Kállai-Szabó, Viktor Fülöp, Géza Jakab, Péter Gordon, Barnabás Kállai-Szabó, Emese Balogh, István Antal

**Affiliations:** 1 Department of Pharmaceutics, Semmelweis University, Hőgyes E. Str. 7, Budapest, 1092 Hungary; 2 Department of Electronics Technology, Budapest University of Technology and Economics, Egry J. Str. 18, Budapest, 1111 Hungary; 3 ViteCer Ltd., Ipartelepi Str. 8/b, Cegléd, 2700 Hungary

**Keywords:** multiple-unit pellet system (MUPS), coating vulnerability, dissolution profile, microfocus X-ray (MFX), micro-computed tomography (microCT)

## Abstract

Most of the commercially available pharmaceutical products for oral administration route are marketed in the tablet dosage forms. However, compression of multiparticulate systems is a challenge for the pharmaceutical research and industry, especially if the individual unit is a coated particle, as the release of the active ingredient depends on the integrity of the coating. In the present study, polymer-coated pellets tableted with different types of excipients (powder, granules, pellets) then were investigated by various tablet-destructive (microscopic) and tablet non-destructive (microfocus X-ray; microCT) imaging methods. The information obtained from the independent evaluation of the *in vitro* drug release profiles model is confirmed by the results obtained by image analysis, regardless of whether X-ray or stereomicroscopic images of the coated, tableted pellets were used for image analysis. The results of this study show that the novel easy-to-use, fast, and non-destructive MFX method is a good alternative to the already used microscopic image analysis methods regarding the characterization of particulates, compressed into tablets.

## INTRODUCTION

Nowadays, oral delivery is still the most common route of administration for delivering active pharmaceutical ingredients (APIs) to the body due to its non-invasiveness and high patient acceptance. Among the various oral dosage forms, tablets and capsules are preferred in the pharmaceutical industry because the administration of these pharmaceutical compositions is convenient, characterized by high physico-chemical stability, and is considered cost-effective (1, 2). As of October 2020, more than 53% of all registered pharmaceutical products in Hungary are tablets, which shows the popularity and wide-spread use of this dosage form (3).

Multiple-unit pellet systems (MUPSs) come in two forms, encapsulated and tableted modified release pellets. The concept of oral multiparticulate dosage forms (tablets or capsules) lies in the idea that the active pharmaceutical ingredient (API) is divided into multiple, individual particles, which distribute evenly in the gastro-intestinal system. Although they are more troublesome to make than single-unit dosage forms, numerous physiological, therapeutic, and technological advantages exist to incentivize this design, for example the “dose dumping” effect can be avoided, and also most of the mucosa’s irritation (4–6). The technological advantages of MUPSs are that incompatible drugs can be incorporated into a single dosage form. Dosage flexibility or tailor-made drug delivery can be provided by the combined use of particles characterized by different amounts of the drug or various rates of drug release. Furthermore, in the case of dysphagia, administration of a controlled-release, single-dose formulation, which is usually a large, non-halved, or non-crushed tablet, reduces patient compliance. Due to the small size of the particles and their unique drug release system, the modified release MUPSs can be halved or even sprinkled, thus providing an attractive and novel formulation, especially in paediatrics and elderly patients (2, 7–9).

While filling pellets in a hard gelatine capsule might seem an easier task to achieve, there is also a growing interest in compressed MUPS, as this formulation cannot be tampered with afterward (Tylenol tampering case; 1982) and the use of gelatine of animal origin can be avoided. They are also easier to swallow compared to the capsule (10–12). Although the compression of multiparticulates has several advantages, its tableting is challenging, especially if the subunits have a functional polymer coating (13–15). To compress a MUPS, the optimal tableting conditions (tableting machine, speed, force, *etc.*) need pairing with the optimal formulation, the latter being complicated due to the difference in characteristics (mainly flowability, bulk density, moisture content, and surface parameters) between the drug-loaded pellets and the tableting excipients (16–19).

Additionally, if the excipients used for compression are not soft or brittle enough (thus not being able to “cushion,” to protect the pellets from pressure), the coated subunit particles may deform while tableting, and so lose the properties set by the coating (20, 21). Multiple approaches have long since been made to overcome these obstacles; most studies focus on finding the ideal cushioning particles (10). These efforts, while trying to avoid segregation with the application of larger tableting excipient particles (granules or pellets), also use a range of materials: from a special type of microcrystalline cellulose (MCC) (22), through additional excipients: from isopropyl alcohol (23) and polyethylene glycol (24) through lactose (12) or stearic acid (25) to wax (26, 27). Another approach is to use a coating that is resistant to the high pressures exerted in the tableting machine; polyvinylpyrrolidone (28), polyvinyl acetate (29), and, surprisingly, metoprolol (30) have been used. There are possibilities in adapting the manufacturing processes as well, like using freeze-drying to produce a porous structure (31), adding an extra granulated layer on top of the coating (32, 33), or utilizing additive manufacturing (34–36).

In the case of a tableted MUPS, it is crucial to check the integrity of the subunits after compression. This can be done *via* multiple methods; besides *in vitro* dissolution studies, the combination of microscopic imaging techniques (be it stereo- or electron microscopy) and the subsequent image analysis (37) are the most wide-spread. However, to be able to study a MUPS non-destructively, one has to search for other, more elaborate methods: spectral imaging techniques (38), terahertz pulsed imaging (TPI), or computed microtomography (microCT). MicroCT, like computer-aided tomography (CAT) devices used in the hospital, utilizes X-rays, and has a much better resolution (voxel size: between 1 and 50 μm), thus being suitable for examining small samples. The microCT technique has already been successfully used to study and characterize the internal structure of several solid pharmaceutical dosage forms such as granules, tablets, micro- and nanoparticles, films, implants, and lyophilized tissue engineering scaffolds (39–42).

In our previous study, we successfully applied microfocus X-ray (MFX) (43) to study the distribution of potassium chloride (KCl) containing polymer-coated pellets as well as the pellet content of the multiparticulate dosage form, while changes in the shape parameters of the pellets have not been studied by MFX. In the present study, we aim to see using various imaging methods (MFX; stereomicroscopic imaging) how the main shape parameters of coated pellets change during compression with various types of filler materials (powders, granules, pellets). Furthermore, whether there is a correlation between the coated, compressed pellets shape parameter data obtained with either MFX technique or from stereomicroscopic images and the dissolution profile of different multiparticulate formulations.

## MATERIALS AND METHODS

### Materials

Ph. Eur. 8th grade potassium chloride was purchased from Molar Chemicals Kft. Hungary. Microcrystalline cellulose (Avicel© PH105 and PH-302) was obtained from FMC Corp., USA. Dimethicone was purchased from Wacker Chemie AG, Germany. Aerosil®200 and Eudragit NE 30 D acrylic film-forming polymer were obtained from Evonik Industries AG, Germany. Micronized talc and indigo carmine were procured from Sigma-Aldrich Chemie GmbH, Germany. Ethanol (96%) as a cosolvent was purchased from Molar Chemicals Kft, Hungary. Silicone emulsion of SE2 type was obtained from Wacker Chemie AG, Germany. The W/O emulgent (Labrafil©M 1944 CS) was ordered from Gattefossé, Lyon, France, and hydroxypropyl-methylcellulose with a nominal viscosity of 3 mPA*s (HPMC; Benecel© E3 Pharm.) was obtained from Ashland Inc., Kentucky, USA. Deionized water was gained from a Christ-Ministil© P-24 ion exchange column (Ovivo Water Ltd., Wolverhampton, UK).

### Preparation of Multiple-Unit Pellet Systems (MUPS)

#### Preparation of Potassium Chloride-Containing Coated Pellets

##### KCl-Loaded Matrix Cores

Potassium chloride-containing coated pellets were made according to the process published and described earlier (44). Briefly, potassium chloride (84.83% *w*/*w*; particle size less than 100 μm), microcrystalline cellulose (15% *w*/*w*), and colloidal silicon dioxide (0.17% *w*/*w*) were pelleted in a pilot-sized rotofluid granulator (Glatt GPCG 15; Glatt GmbH; Germany). The batch size was 30 kg. The colloidal silicon dioxide is used to avoid the clumping of potassium chloride crystals. As a pelletizing liquid, a dimethicone emulsion (2.0% *w*/*w*) was used, which was sprayed onto the powder mixture with a tangential spray gun. The process parameters were as follows: fluidization airflow = 650–1000 m^3^/h; roto speed = 250–450 rpm; inlet air temperature = 20–22°C; spray rate = 100–200 g/min; atomization air pressure = 2.5 bar. The wet pellets were dried (drying temperature = 80°C) in the same apparatus and then the dry particles were sieved. A sieve fraction between 800 and 1600 μm was used for the coating process.

##### Coating of KCl-Loaded Pellets

During the coating process, 180 g of acrylic polymer dispersion was sprayed onto 400 g of KCl-loaded matrix pellets using a configured fluidized bed apparatus (Aeromatic Strea I, Aeromatic-Fielder AG, Switzerland). The nozzle diameter was 0.8 mm. In preparing the polymer dispersion, the dye (0.02% *w*/*w*) was first dissolved in the water/alcohol (21.23% *w*/*w* and 10% *w*/*w*) solution. Micronized talc (1.78% *w*/*w*), silicone emulsion (3.43% *w*/*w*), and Eudragit NE 30D (40% *w*/*w*) were then added and finally supplemented with water (23.54% *w*/*w*). The polymer dispersion was stirred continuously to prevent sedimentation of insoluble particles. The process parameters were as follows: inlet and outlet temperature = 20–28°C; atomizing air pressure = 0.8 bar; fluidization airflow rate = 70–90 m^3^/h; spray rate = 1–4 ml/min.

#### Preparation of Fillers

- For Filler A—microcrystalline cellulose (MCC) powder—original form was used.
- For Filler B—the MCC granules—100.0 g of binding agent, containing 10% (*w*/*w*) water solution of hydroxypropyl methylcellulose (HPMC) was added to 200.0 g of Avicel PH-302, then mixed for 10 min at 600 rpm, then for 6 min at 900 rpm in a Universal Machines UMC-5 mixer (Stephan Machinery GmbH, Hameln, Germany), while adding another 15.0 g of deionized water.
- For Filler C—the cushioning pellets—65.0 g of Labrafil© M 1944 CS and 35.0 g of deionized water were added to 200.0 g of Avicel PH-302, then homogenized with aforementioned Universal Machines UMC-5 mixer before extrusion with a Caleva Multi Lab extruder (Caleva Process Solutions Ltd., Sturminster Newton, UK) at 120 rpm through die holes of 1 mm. The extrudates were subsequently rounded in a spheronizer (Locost GSZF-AK spheronizer; Locost Kft., Tiszaalpár, Hungary), at 1000 rpm, for 1.5 min.
- For Filler D—the MCC pellets—160 g of deionized water was added to 200 g of Avicel PH-302, then processed with using the same steps as Filler C.

All wetted samples were dried in an Aeromatic Strea-1 fluid bed (Aeromatic AG, Bubendorf, Switzerland) at 100 m^3^/h air flow rate, with the air intake temperature set to 60°C, until the air outtake temperature was the same. The samples were then fractioned on a Retsch AS 200 vibration sieve (Retsch Gmbh, Haan, Germany) at an amplitude of 1.5 mm, and the 800–1250 μm-sized fractions were used.

##### Compression of Multiple Unit Systems

Two hundred fifty grams of each ratio of 9:1 filler A, B, C, or D and potassium chloride-coated pellets were mixed in a cube mixer (AR400, Erweka GmbH, Heusenstamm, Germany) for 10 min, at 25 rpm. All four blends were tableted on a rotary tableting machine with oblong punches, (KMP-8, Kambert Machinery Co., Vatwa, India) at 7.5 rpm, while tablet mass was set around 800 mg. The applied compression force was set to gain multiple unit systems with the highest tensile strength possible. This produced tablets T_A_, T_B_, and T_C_ containing Filler A, B, and C, respectively. Filler D, due to its hardness, could not be tableted, as the pellets showed no sign of plastic deformation.

### Characterization Methods

#### Physical Characterization of Particles

##### Shape and Size Analysis

Image analysis of KCl-loaded coated pellets and filler particles was carried out before compression. One hundred fifty pellets or granules were randomly chosen from each batch to be analyzed. Photomicrographs of particles (granules or pellets) were taken with a digital camera (Coolpix 4500, Nikon, Tokyo, Japan) connected stereomicroscope (SMZ 1000, Nikon), with an image resolution of 12.8 μm/pixel, and then analyzed using image processing software (ImageJ 1.48v, Wayne Rasband, National Institute of Health, USA). The investigated particles were illuminated from the top applying a cold white coherent fibre-optic light (230 V, 185 W, 50/60 Hz, the diameter of bundle 5.4 mm) of halogen light source (Intralux 5000–1 type,Volpi, Switzerland). Shape aspect ratio (AR), the ratio of the maximum Feret diameter and the minimum Feret diameter (perpendicular to the maximum Feret diameter), was used for the characterization. The values presented for each type of investigated batch are the average and standard deviation (SD) calculated from the measurement of 150 individual particles.

Ten pieces of each filler material were then fixed on a sample holder using double adhesive tape, then gold coating was applied with an Emitech K550X Sputter Coater (Quorum Technologies Ltd., Ashford, UK) for 2 min. Examinations were performed by means of a scanning electron microscope (FEI Inspect S50) at 20.00 kV accelerating voltage. Working distance was between 21 and 22 mm. Original magnification was 300–4000× with an accuracy of ± 2%.

##### Tensile Strength of Particles

Plasticity and breaking force of Filler B, C, and D were determined by a texture analyzer (CT-3, Brookfield Engineering Laboratories, Middleborough, USA) operating with a 4.5-kg load cell in compression mode. Ten pieces of each sample were placed individually on a ceramic plate and tested, at a test speed of 0.05 mm/s, trigger load of 0.05 N, and data rate of 5 points/s. During the measurement, a force-distance curve was recorded by TexturePro CT v1.4 software (Brookfield Engineering Laboratories, Middleborough, USA). The fracture force (*F*) and the diameter (*d*) of each individual pellet/granule were recorded, from which the tensile strength (*σ*_*f*_*(s)*) of particles was determined using the following equation: (45).

1 $$ {\sigma}_f(s)=\frac{1.6\times F}{\pi \times {d}^2} $$

Flowability, bulk density, and Hausner ratio (HR) of 100 g of Fillers A, B, and C, as well as potassium chloride-containing coated pellets were measured according to European Pharmacopoeia 9th edition. The following equipment was used for the measurements: Pharmatest PTG (Pharmatest Apparatebau AG; Germany) for flowability, and a STAV 2003 Stampfvolumeter (J. Engelsmann AG., Germany) for the bulk density and Hausner ratio.

##### Physical Characterization of Tablets Containing KCl-Loaded, Coated Pellets

The individual tablet mass (Sartorius LA 230S, Sartorius AG, Goettingen, Germany), dimensions (Mitutoyo Absolute, Mitutoyo Corporation, Kanagawa, Japan), tensile strength (Pharmatron 8 M, Pharmatron AG, Thun, Switzerland), and friability of tablets (Erweka AR, Offenbach/Main, Germany) were measured in compliance with Ph. Eur. 9. The *in vitro* disintegration time of the tablets was determined by a Pharmatron DISI 2 tester (Pharmatron AG, Thun, Switzerland), also according to Ph. Eur. 9. The number of samples for each test was 20 tablets for individual tablet mass, 10 tablets for dimensions and tensile strength, 10 tablets also for friability, and 6 tablets for *in vitro* disintegration time test.

#### Dissolution Study of Prepared MUPS

The *in vitro* dissolution studies were carried out using the USP certified dissolution apparatus I (basket method; Hanson SR8-Plus™ Dissolution Test Station with E-probe; AutoPlus Maximizer and Multifill collector, Hanson Research Corp., Chatsworth, USA). The volume of the dissolution medium (deionized water) was 450 ml per vessel maintained at a temperature of 37 ± 0.5°C. The spindle rotation rate was adjusted to 100 rpm. The test was carried out for 14 h, filtered samples (10 μm; Hanson Research Corp., Chatsworth, USA) being taken automatically at 15, 30, 60, 90, and 120 min, then every 2 h after, with media replacement. The result was the average of six measurements. To determine the amount of drug released, a HI4107 Chloride Combination Ion Selective Electrode attached to a HI902 Automatic Potentiometric Titration System (Hanna Instruments Inc., Woonsocket, USA) was used, operating in ISE mode. The ionic strength of each sample was adjusted with 1 ml of HI4000 ISA solution. To compare the resulting dissolution data, *f*_1_ and *f*_2_ values were calculated according to the following formulae:

2 $$ {f}_1=\left(\ \frac{\sum_{t=1}n\left|{R}_t-{T}_t\right|}{\sum_{t=1}n{R}_t}\ \right)\times 100 $$

3 $$ {f}_2=50\times \log \Big\{{\left[1+\left(1/n\right){\sum}_{t=1}n{\left({R}_t-{T}_t\right)}^2\right]}^{-0.5}\times 100 $$

where *R*_*t*_ and *T*_*t*_ are the cumulative percentage dissolved at the selected time points of the reference and the test product (46).

##### Investigation of Potassium Chloride-Loaded, Coated Pellets After Compression

Compressed KCl-loaded pellets were investigated by various imaging methods and image analysis. Tablets (T_A_, T_B_, and T_C_) were investigated by both destructive and non-destructive methods.

MUPSs of each tablet type were disintegrated *in vitro* to isolate at least one hundred fifty individual KCl-loaded, coated pellets after compression. Image analysis of the potassium chloride pellets obtained in a non-destructive manner for the tablet was performed with a camera-connected stereomicroscope (Nikon SMZ 1000) and software as described above, image resolution being 11.9 μm/pixel. KCl-loaded, coated pellets exposed to tableting were also subjected to scanning electromicroscopic images, which also used the equipment already mentioned above. Tablets of each type (T_A_, T_B_, and T_C_) were placed on a metal plate, then their X-ray images were captured by a DAGE XD 6600 X-ray inspection system (Nordson-DAGE, Avlesbury, UK). Tube power was 1.00 W, tube voltage 100 kV, and image resolution 92 pixels/mm. Twelve tablets of each type were captured, among which at least 40 pellets (of each tablet type) were isolated digitally. These images were then also analyzed using the image analysis software mentioned above (ImageJ) to determine the shape and size characteristics of KCl-loaded, coated pellets after compression. Statistical analysis was performed using STATISTICA 13.4.0.14 software (TIBCO Software Inc., Palo Alto, CA, USA).

Three tablets of each type (T_A_, T_B_, and T_C_) were mounted and scanned with a micro-computed tomography (microCT) system (VTomEx-s, Phoenix|x-ray, a GE Healthcare Company, Little Chalfont, Buckinghamshire, UK). The images consisted of 960 slices with a voxel size of 81.128 μm in all three axes. The scaffolds were imaged and re-constructed in three dimensions. Regions of interest (ROI) of the same size were re-constructed and analyzed using microCT with the same thresholds.

## RESULTS AND DISCUSSION

### Physical Characterization of Particles

Due to the plastic nature of MCC, it is often used as an excipient for both tablet compression and pelletizing processes. The function of the various tableting filler for the preparation of tableted MUPS is complex. They must fill the space between the particles, act as a cushioning agent, *i.e.,* dampen the effect of the compressive force occurring through the tableting process. It is also necessary to form a uniform blend with the particles during tableting. Even with a relatively low compression force, the inert excipient must produce a sufficiently hard tablet, which is characterized by a rapid disintegration time and has no effect on the release of the active ingredients of the particles (14).

**Fig. 1:**
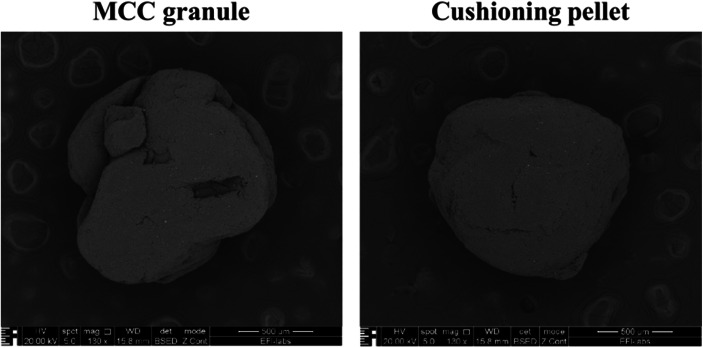
Scanning electron microscopic images of produced particles used in compression

The process parameters were found to be acceptable for producing the filler particles (granules, pellets) used in this study. Table I summarizes the characteristics of the potassium chloride-loaded, polymer-coated pellets and Fillers A–D. It follows from the definition of a pellet that the particles must have close to spherical geometry. According to publications, the maximum acceptable value of the aspect ratio as a shape parameter can be 1.20. (47, 48). The starter KCl pellets came closest to the ideal aspect ratio of 1.00 (which value means perfect roundness), while the novel excipient-based filler pellets (Filler C and D) proved to be acceptably rounded as well. Filler B (granules), as expected, were the least rounded (showing the highest AR value) of all the examined materials. On the SEM-image (Fig. 1) showing the produced MCC based granule (Filler B) and cushioning pellet (Filler C) before compression, there are visible differences in shape between the two particles, which support the results of the stereomicroscopic image analysis. The granule is irregularly shaped, and the comprising particles are visible. In contrast, the pellet, as most MCC pellets, is rather spherical, and its surface is even. Provided that MCC powder is not comprised of aggregated particles, no shape examination was carried out.

**Table I:** Physical Characteristics of Tableting Materials

Material		Aspect ratio (*n* = 150; mean ± SD)	D_Feret Max_ (μm) (*n* = 150; mean ± SD)	Tensile strength (N/mm^2^) (*n* = 10; mean ± SD)	Flowability (g/s*)* (*n* = 3; mean ± SD)	Bulk density (g/cm^3^)	HR
KCl pellet		1.04 ± 0.03	1242 ± 190	3.96 ± 0.92	14.1 ± 0.2	1.28	1.01
MCC-based filler	A (powder)	n. m.	n. m.	n. m.	5.1 ± 0.1	0.46	1.26
	B (granules)	1.45 ± 0.23	1450 ± 310	1.08 ± 0.80	7.3 ± 0.1	0.77	1.04
	C (pellet)	1.14 ± 0.18	1392 ± 253	n. d.	6.6 ± 0.1	0.70	1.06
	D (pellet)	1.12 ± 0.08	1315 ± 130	6.69 ± 3.81	12.3 ± 0.1	0.97	1.03

*n. d.*, not determined; *n. m.*, not measured

The inert excipients in the form of powders (49, 50), granules (51, 52) or pellets (26, 53) are also used as fillers for tableting pellets in the literature. The produced pellets were found to be very similar in size, characterized by a low SD value, suggesting a narrow particle size distribution. As to the mean size of the particles, the starter KCl pellets proved to be the smallest (with a maximum Feret diameter of 1242 μm), then the produced excipient-based fillers were in the following order: Filler D (pellet) – Filler C (pellet) maximum Feret dimaters of 1315 and 1392 μm, respectively. Of the produced particles, the granules (Filler B) are characterized by the largest particle diameter (1450 μm) and a higher SD value (310 μm) resulting from the production.

**Fig. 2:**
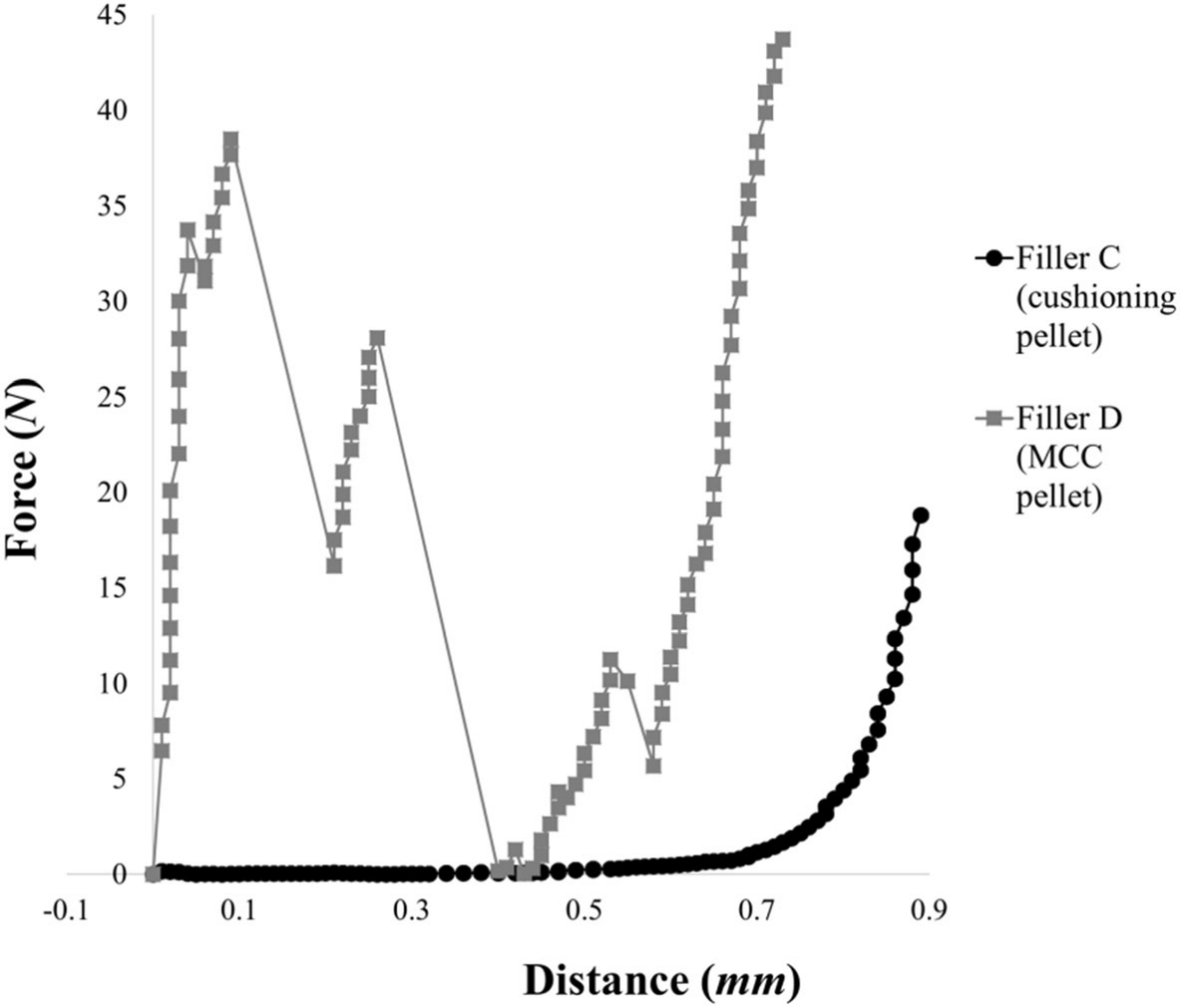
Representative force-distance curves of produced pellets

The tensile strength of the materials was in the following order: Filler D–KCl pellets–Filler B, from high to low. As of Filler C, no fractures were recorded during the measurement—this, along with the results of Filler D, is shown on Fig. 2 (force–distance curves). Among the fillers produced, the granulate, which was not made by extrusion, can be characterized by a looser structure, so that its breaking strength is lower than that of pellets. In the case where surfactant was also used in the extrusion of MCC, it significantly reduced the breaking strength. A similar finding was found by Nikolakakis *et al*. when producing MCC-based pellets containing the active ingredient, oil/surfactant by extrusion (54). As in the previous study, in the case of Filler C, the interparticular MCC bonds were weakened, resulting in a decrease in the breaking strength of the Filler C pellet.

The flow rates of the different particles were as follows: KCl and MCC pellets flowed the fastest, while Filler A flowed the slowest. The flow rate of Filler C was in between. These results correlate well with the particles’ shape characteristics, the only exception being Filler C, whose flow was most likely affected by its plasticity. The bulk density measurements showed the following results: the KCl pellets had the highest density, while Filler A the lowest, also condensing the most. The rest of the materials showed only smaller differences.

### Physical Characterization of Tablets

Table II summarizes the results of the physical examinations; T_A_ and T_B_ tablets had unacceptable friability, the former failing the mass uniformity examination as well, which means T_A_ and T_B_ tablets were deemed unacceptable by Ph. Eur. 9. T_C_ tablets, however, fulfilled the requirements. Note that despite the addition of oil into the formulation, the disintegration time did not exceed 4 min, which is, while slower than the other formulations, still well within the acceptable range (disintegration time less, than 15 min).

**Table II:** Physical Characteristics of MUPSs

	T_A_ tablet	T_B_ tablet	T_C_ tablet
Tablet mass (mg) (*n* = 20; mean ± SD)	839 ± 64	815 ± 20	792 ± 20
Tablet height (mm) (*n* = 10; mean ± SD)	7.5 ± 0.2	6.1 ± 0.1	6.3 ± 0.1
Tablet diameter (mm) (*n* = 10; mean ± SD)	16.6 ± 0.03	16.6 ± 0.04	16.5 ± 0.1
Tablet hardness (N) (*n* = 10; mean ± SD)	68 ± 11	37 ± 4	30 ± 5
Friability (%)	not accepted	not accepted	0.22
Longest disintegration time (s)	6	10	186

### Dissolution Study of Prepared MUPS

**Fig. 3:**
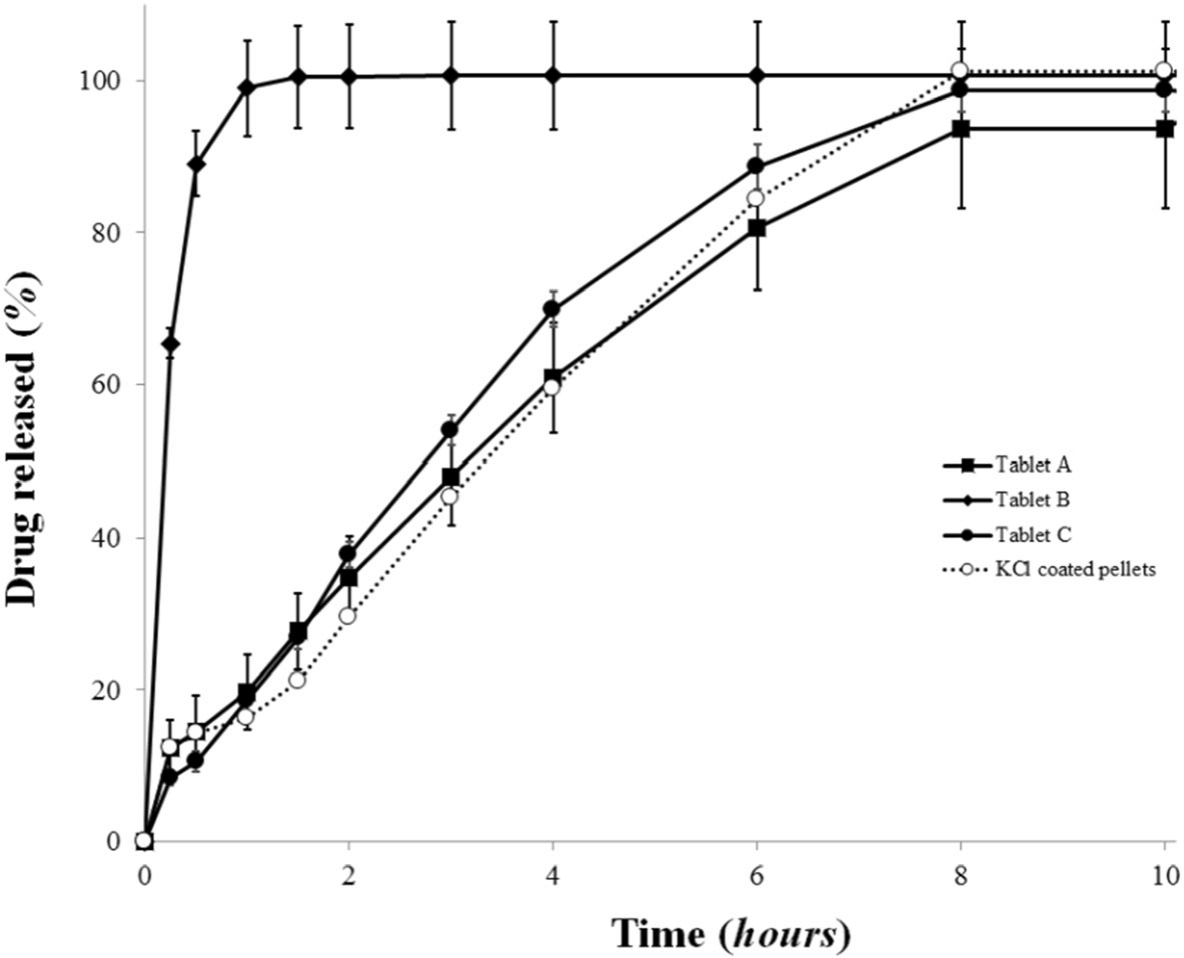
KCl release profile of uncompressed pellets (○) and various types tableted pellets (T_A_ (■), T_B_ (□), and T_C_ (●) tablets); (mean ± SD; *n* = 6)

The results of the 10 h dissolution tests are shown on Fig. 3. The T_A_ and T_C_ tablets produce comparably the same results as the API pellets on their own. Furthermore, it can be seen that for the first sampling point (at 15 min), the amounts of KCl released from tableted MUPSs were almost the same regardless of the type of filler. Consequently, it can be concluded that tablet disintegration during dissolution is not a rate-determining step, which is a requirement for tableted, rapidly disintegrating multiparticulate systems (14). The fact that T_B_ tablets release the drug almost instantly is due to the fractures suffered by the coating during the tableting process, which in turn are caused by the filler material (the MCC granules) being hard and brittle. Both the MCC powder and the MCC cushioning pellets were able to produce tablets with the API pellets intact; however, only the latter could achieve an acceptably similar drug release, with *f*_1_ and *f*_2_ values being 10.92 and 63.10. *f*_1_ and *f*_2_ values for tablet A were as follows: 10.6 and 50.94.

### Investigation of Potassium Chloride-Loaded, Coated Pellets After Compression

**Fig. 4:**
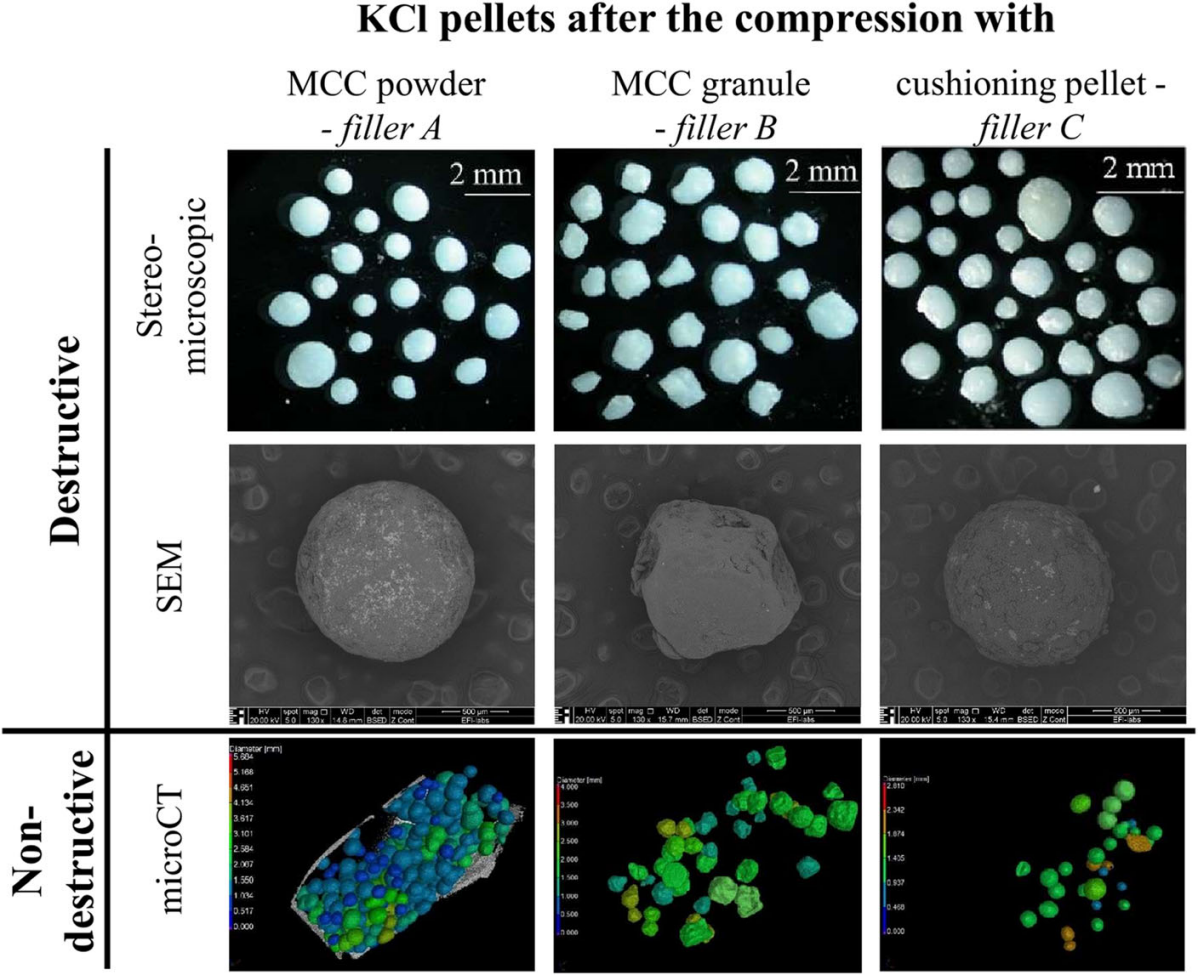
Morphological characteristics of KCl-loaded, coated pellets after compression. A: from T_A_ tablets, B: from T_B_ tablets, C: from T_C_ tablets

**Fig. 5:**
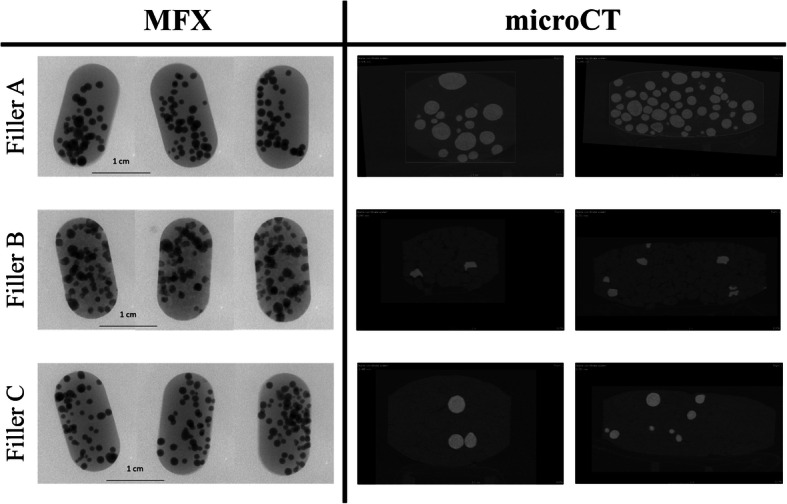
MFX and microCT scans of T_A_, T_B_, and T_C_ tablets

**Fig. 6:**
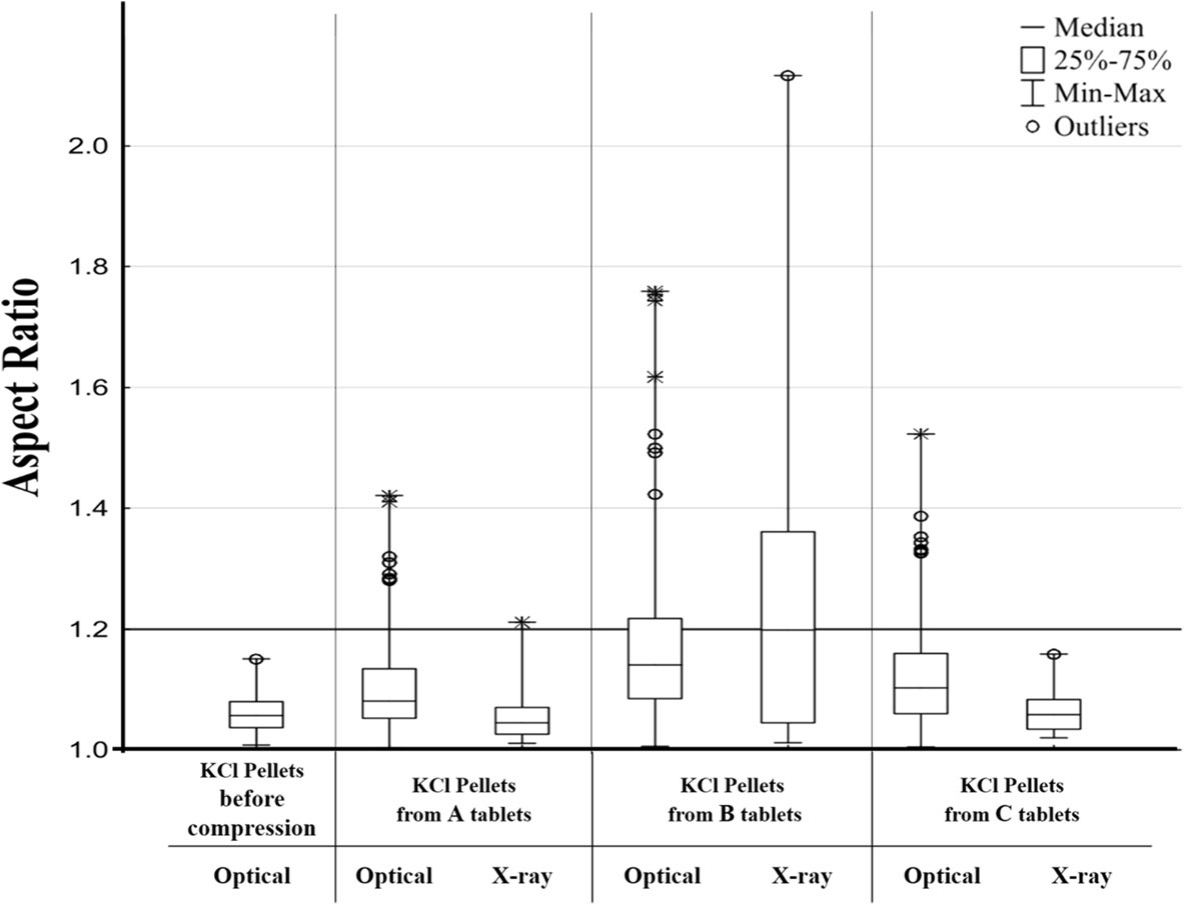
Box-plot of analysis from images produced by different techniques (optical = stereomicroscopic and MFX)

Figure 4 contains stereomicroscopic and SEM images of KCl-loaded coated pellets after compression. To capture these, a process non-destructive regarding the pellets, but destructive regarding the MUPS tablets (disintegration) was carried out. The stereomicroscopic images were used for image analysis, and the results are shown in Table III. Upon regarding the AR values, the largest difference before and after compression is shown by those compressed with Filler B (granules). This can also be seen on the SEM images—the pellet from the B tablet is deformed, showing the effect of compression forces. Other imaging methods were also used, which were non-destructive regarding the MUPS tablet: MFX and microCT. Images captured with the former are shown on Fig. 5; the images were also analyzed, the results being also contained in Table III. As the stereomicroscopic data, these also show that the KCl pellets compressed with Filler B (granules) were the most affected by the compression force, their shape deforming to the largest degree. Figure 6 compares the aspect ratio gained from the analysis of images from different sources; both stereomicroscopic and X-ray data show the largest deviation and difference between minimum and maximum values in the case of Filler B among their respective counterparts. Additionally, Table III displays the ratio of the maximum and minimum Feret diameter of the particles (gained from both the stereomicroscopic and the electron microscopic data), while the starter pellets’ value is 1.078, indicating a near spherical shape, the pellets from the B tablets have been deformed, demonstrated by values of 1.210 and 1.271. The same results are also shown on the microCT images, on both Fig. 4 and Fig. 5. The presented data will clarify that in the case of compressed pellets, the dissolution tests are confirmed by the results of image analysis performed with different imaging methods. Moreover, MFX can also be used for image analysis, examining the shape parameters of pellets.

**Table III:** Shape Parameters of KCl-Loaded, Coated Pellets Before and After Compression

Origin of images	Origin of pellets	Mean ± SD of pellet aspect ratio	D_Feret Max_/D_Feret Min_
Stereomicroscope	KCl pellets before compression	1.06 ± 0.03	1.078
	KCl pellets from T_A_ Tablets	1.105 ± 0.07	1.124
	KCl pellets from T_B_ Tablets	1.174 ± 0.13	1.210
	KCl pellets from T_C_ Tablets	1.133 ± 0.12	1.105
MFX	KCl pellets from T_A_ Tablets	1.058 ± 0.04	1.098
	KCl pellets from T_B_ Tablets	1.246 ± 0.14	1.271
	KCl pellets from T_C_ Tablets	1.063 ± 0.03	1.121

## CONCLUSION

In the present study, tableting of prepared KCl-loaded, acrylic polymer-coated pellets was performed using various MCC-based excipients (powder, granules, pellets). The main characteristics of produced particles were examined before the compression (shape and size analysis, hardness, *etc.*). We searched for a relationship between the results obtained in standard tablet tests (hardness, wear loss, dissolution profile, *etc.*) and the values obtained by computer image analysis, and whether the MFX technique is suitable for determining the shape parameters of pellets within a tablet. To this end, we also performed stereomicroscopic examinations and 3D microCT measurements. Both the differences between the dissolution curves and the results of image analysis with various imaging techniques reflect the damage or possibly integrity of the individual coated pellets during compression. Thus, the results support that the novel MFX method is an alternative to the already frequently used microscopic image analysis methods for the characterization of particles compressed into tablets. Furthermore, MFX is an easy-to-use, fast, and non-destructive method is conceivable to use it as a process analytical technology (PAT) that is extremely important in pharmaceutical manufacturing today.
